# A case of severe unprovoked hemorrhage in an elderly male: a case report

**DOI:** 10.1002/ccr3.1029

**Published:** 2017-06-01

**Authors:** Petros Ioannou, Emmanouela Tsagkaraki, Constantinos Tsioutis, Maria Devetzoglou, Irene Xylouri, Achilleas Gikas, Symeon Panagiotakis

**Affiliations:** ^1^ Internal Medicine DepartmentUniversity Hospital of HeraklionHeraklionCreteGreece; ^2^ Hematology DepartmentUniversity Hospital of HeraklionHeraklionCreteGreece

**Keywords:** Acquired hemophilia, bleeding, case report, coagulation, factor VIII

## Abstract

Acquired hemophilia is a rare but potentially fatal clinical condition requiring clinical suspicion to reach to a diagnosis, especially in elder patients. This diagnosis should be suspected in patients that present with unexplained persistent bleeding from skin, soft tissues, and mucosa and have a prolonged aPTT.

## Introduction

Acquired hemophilia is a rare but life‐threatening disorder due to acquired inhibitors of coagulation, most commonly against factor VIII, even though inhibitors against other factors, namely II, V, VII, IX, XI, and XIII, have also been reported 1, 2. Its incidence is age‐related with more than 80% of the patients being older than 65 years at diagnosis 3. Diagnosis is usually set in a patient who presents with unprovoked bleeding, most commonly in soft tissues. Although the disorder is associated with autoimmune diseases, solid tumors, or hematologic malignancies, in about 50% of cases no cause can be found 1. Severe bleeding may develop in more than 80% of patients, with a fatal outcome in up to 22% 1. Treatment includes administration of coagulation factors, such as factor VIII, recombinant activated factor VII or activated prothrombin complex concentrate, and immunosuppressive therapy with corticosteroids and other medications such as cyclophosphamide or rituximab 4, 5.

## Case Presentation

**Figure 1:**
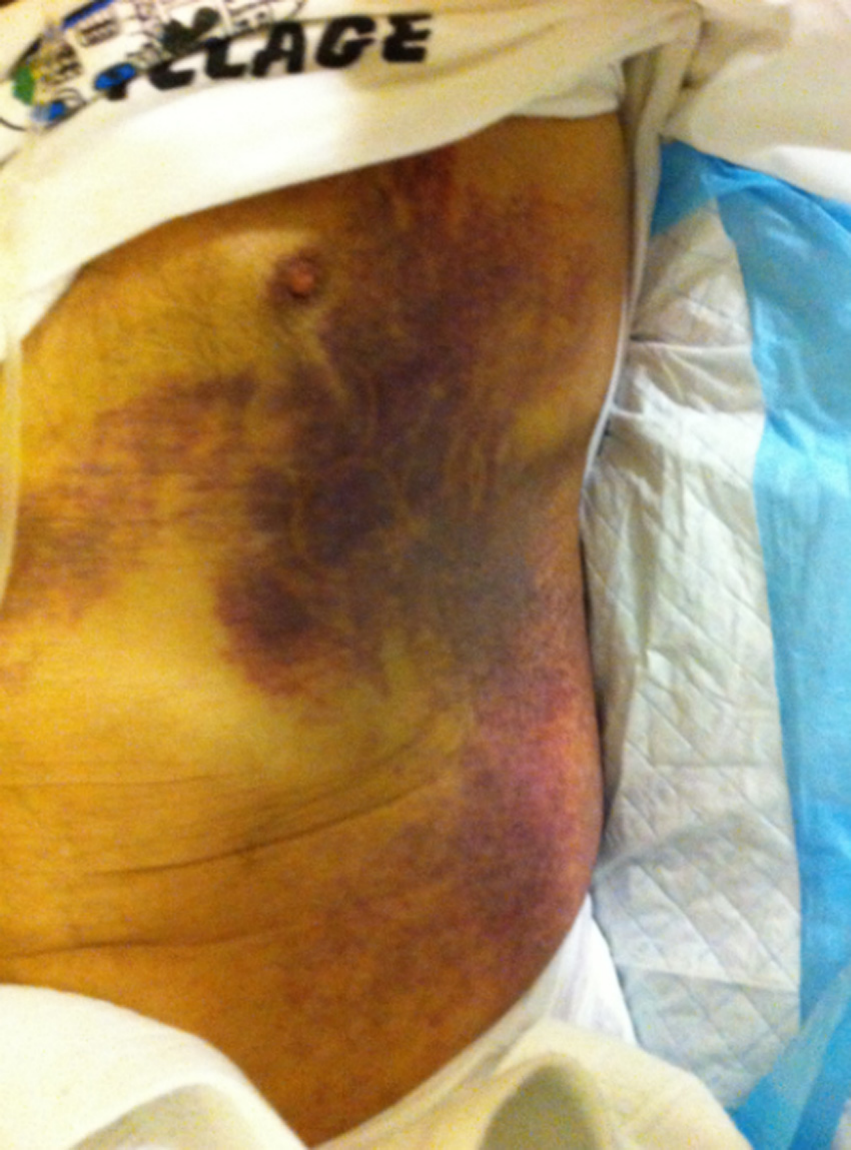
Evolution of a small ecchymosis near the left ear to a large hematoma reaching the sternum and the left iliac crest.

We present the case of a 77‐year‐old Caucasian male who presented to our hospital complaining of an unprovoked ecchymosis behind his left ear without any previous hemostasis dysregulation or treatment with anticoagulants or NSAIDs. His past medical history included osteoporosis, excessive alcohol intake that was discontinued 6 months before his presentation, and active smoking. Upon examination, he was pale and had a small ecchymosis behind his left ear. His laboratory tests revealed a hypochromic anemia, with a hemoglobin of 7.7 g/dL (reference 12–15.5 g/dL), an increased aPTT count of 121 sec (reference <36 sec), and a normal prothrombin time. Lupus‐anticoagulant test and antiphospholipid antibodies were found negative and mixing studies were performed (Tables 1 and 2), showing correction of aPTT at time zero but prolongation at 1 and 2 h of incubation, while the coagulation assays revealed the presence of an acquired inhibitor of factor VIII (Table 2), while factors IX and XI were not affected and antiphospholipid antibodies were not present, setting the diagnosis of acquired hemophilia. To diagnose a possible underlying disorder, computed tomography of the chest and abdomen, gastroscopy, colonoscopy, and bone marrow biopsy were performed, with no significant findings. Cancer serum markers, virology, and immunology for vasculitis, systemic lupus erythymatosus and rheumatoid arthritis were negative. The patient's condition deteriorated due to continued bleeding, with the ecchymosis behind his left ear evolving into a hematoma reaching the sternum and the left iliac crest (Fig. 1), and he also manifested melenas due to gastric bleeding. He was transfused with twelve units of packed red blood cells in total, while activated factor VII and activated prothrombin complex concentrate (IX, II, VII, and X) were administered so to restore the aPTT and control the bleeding. Immunosuppression was started with three daily intravenous pulses of methylprednisolone, followed by prednisolone and cyclophosphamide, while intravenous immunoglubulin was given for five consecutive days. The patient's hemoglobin was stabilized, his aPTT decreased, his general condition improved, and he was discharged on day 47 with a gradual taper of the corticosteroids and cyclophosphamide for the next 3 months. In a follow‐up visit, aPTT prolongation and a new episode of superficial bleeding were noted, and corticosteroids were restarted. During 2 years of follow‐up and a repeat thorough workup, no underlying disorder has been found.

**Table 1:** Mixing reactions to establish the diagnosis of acquired hemophilia

Mixing Study	Patient's aPTT (sec)	Control aPTT (sec)	aPTT of the 1:1 mixture (sec)
No incubation	131.7	26.1	68.7
One‐hour incubation	129.3	32.5	109.6
Two‐hour incubation	133.7	46.1	141.3

**Table 2:** Mixing reactions establishing the presence of inhibitor of factor VIII

	No incubation		One‐hour incubation	
	aPTT (sec)	Factor VIII (%)	aPTT (sec)	Factor VIII (%)
Patient	121.1	0	111	0
Control	24.4	118	27.4	103
Mix	77.6	0.6	111.2	0

The first and third columns show the aPTT of the patient, the control, and their 1:1 mixture without incubation or after one‐hour incubation. The second and fourth columns demonstrate the percentage (%) of the coagulation factor VIII activity assessed by the aPTT of the 1:1 mixture of the patient's, the control's, or their mixture's sample, with a sample from a patient deficient in coagulation factor VIII.

## Discussion

In this case report, we describe the case of an elderly male who presented with severe automatic bleeding that was proved to be associated with lack of activity of factor VIII, due to acquired hemophilia, that necessitated significant immunosuppression to be controlled, while a thorough workup did not reveal an underlying factor that could be associated with his presentation.

The diagnosis of acquired hemophilia is set in a patient without a prior diagnosis of congenital hemophilia, who develops bleeding, most commonly in soft tissues, with no or minimum mechanical damage, due to development of inhibitors of coagulation factors, most commonly against factor VIII 1, 2. The etiology of acquired hemophilia A (the most common form of acquired hemophilia, which is due to inhibition of the factor VIII) has been examined through three large studies by Green, Morrison, and Collins, showing that it occurs at about 17% in the context of an autoimmune disorder (such as rheumatoid arthritis, systemic lupus erythematosus, or inflammatory bowel disease) in 6.7–14.7% in the context of a malignant disease (solid such as prostate, lung or colon, or hematologic, like non‐Hodgkin lymphoma, multiple myeloma), in 2–4.5% in the context of skin diseases (such as psoriasis), and up to 11% in the context of pregnancy. Importantly, in about 40–63.3% of cases, no associated factor can be found, so the acquired hemophilia is classified as idiopathic, even though evidence suggests that a diagnosis such as the ones mentioned above can be set even years after the diagnosis of acquired hemophilia 3, 6, 7.

## Conclusion

To conclude, acquired hemophilia is a very rare clinical condition. Its diagnosis requires clinical suspicion, especially in older patients, and sound clinical judgment so not to be confused by other diagnoses of hemostasis. Clinicians should suspect the diagnosis of acquired hemophilia in patients that present with unexplained persistent bleeding from skin, soft tissues, and mucosa and have a prolonged aPTT.

## Consent

Written informed consent was obtained from the patient for publication of this case report and any accompanying images. A copy of the written consent is available for review by the editor in chief of this journal.

## Authorship

PI, CT, MD, IX and SP: were actively involved in the clinical care of the patient. PI, ET, CT and MD: wrote the manuscript. IX, AG, and SP revised the manuscript.

## Conflict of Interest

The authors declare that they have no competing interests.
